# Lessons Learned from Probing for Impacts of Triclosan and Triclocarban on Human Microbiomes

**DOI:** 10.1128/mSphere.00089-16

**Published:** 2016-05-18

**Authors:** Rolf U. Halden

**Affiliations:** Biodesign Center for Environmental Security, Biodesign Institute, School of Sustainable Engineering and the Built Environment, and Global Security Initiative, Arizona State University, Tempe, Arizona, USA, and Environmental Chemistry Department, Swiss Federal Institute of Aquatic Science and Technology (EAWAG), Dübendorf, Switzerland

**Keywords:** antimicrobials, antibiotic resistance, body burden, community effects, human exposure, microbiome, triclocarban, triclosan

## Abstract

Despite increasing interest in the effects of triclosan and triclocarban on human biology, current knowledge is still limited on the impact of these additives to antimicrobial personal care products on the human microbiome. A carefully designed recent study published in *mSphere* by Poole and colleagues [A. C. Poole et al., mSphere 1(3):e00056-15, 2016, http://dx.doi.org/10.1128/mSphere.00056-15] highlights both the power of novel methodologies for microbiome elucidation and the longstanding challenge of employing small-cohort studies to inform risk assessment for chemicals of ubiquitous use in modern society.

## COMMENTARY

Over the last decade, two trichlorinated binuclear aromatic antimicrobials, the phenolic compound triclosan [TCS; 5-chloro-2-dichlorophenoxy(phenol]) and the nonphenolic carbanilide triclocarban [TCC; 3-(4-chlorophenyl)-1-(3,4-dichlorophenyl)urea], have come under intense regulatory scrutiny for purported overuse, lack of efficacy, widespread human exposure, and an array of unwanted effects on human health and the environment (reviewed in reference 1). A new study by Poole et al. (2) employed the latest advances in molecular biology to elucidate whether combined use of TCS and TCC in personal care products has a detectable effect on the human gut and oral microbiome, yielding a vast data set that is interesting and instructional in several ways.

Poole et al. (2) employed a crossover control study design in their work, which offers the advantage of each participant serving as his or her own control, a prudent choice in experimental layout in assessing the effects of chemical exposures that are essentially impossible to avoid completely today (3–5). Both antimicrobials are present in over 2,000 different personal care, household, and medical products, ranging from soaps (TCC and TCS) to building materials and toothpaste (TCS) to food packaging (TCS) to medical devices (TCS) (1). Consequently, all possible exposure routes, including absorption (e.g., soaps, toothpaste), ingestion (e.g., drinking water, food), inhalation (e.g., aerosols, dust), and even injection/implantation (e.g., medical sutures and devices), are relevant for TCS/TCC.

Reasons abound to study exposure to TCS/TCC in the context of potential or known adverse human health effects. By 2014, reported outcomes from acute and chronic exposures included irritation of eyes and skin, sensitization to aeroallergens and food, and immunologic reactions such as allergies, developmental and reproductive toxicity, inhibition of muscle function, endocrine disruption, and antimicrobial drug resistance (reviewed in reference 1). New data on human body burdens for TCS and TCC have become available in the past 3 years (3–5), and new reports suggest adverse outcomes, including, for TCS exposure, development and proliferation of cancer cells (6–11), endocrine disruption (8, 12), reduced sperm quality in men (13), and increased risk of obesity (14) and, for TCC exposure of humans, decreased gestational age at birth (5).

Not all of the reported adverse outcomes of TCS/TCC exposure determined in animals are relevant to humans, and the unwanted effects observed in high-dose animal experiments can seldom be observed in cohorts of humans, who experience much lower environmentally relevant exposures. And since small-cohort studies are notorious for featuring limited power, observations made among small cohorts limited in the number of study participants (see, e.g., reference 5) may or may not be reproducible in larger, follow-up investigations.

Association between exposure to TCS/TCC and human microbiome alterations, while expected, may be difficult to demonstrate, with the oral microbiome offering the best prospect of success. Prior to the work by Poole et al. (2), no mammalian studies had been conducted to elucidate specifically the impact of TCS and TCC on the gut microbiome. A rare study examining the effects of TCS exposure at low, environmentally relevant levels on the gut microbiome of fathead minnows (*Pimephales promelas*) found rapid, significant alterations following exposure, with detectable perturbations in alpha and beta diversity that proved to be short-lived and reversible (15). A study on the bacterial communities extant in embryos of zebrafish (*Danio rerio*) found interactive effects from coexposure to TCS and UV radiation (16). An examination of the human nasal microbiome showed a positive correlation between exposure to TCS and the occurrence of *Staphylococcus aureus* in nasal secretions (17). The most comprehensive body of work on the effect of TCS on human microbiomes has been performed on the oral cavity, motivated by reports of TCS acting as an antigingival agent limiting periodontitis. A double-blind, prospective, crossover randomized study examining the efficacy of mouth rinse containing TCS as one of a total of three active ingredients found significant (23.8% to 46.9%; *P* < 0.001) reductions in parameters for regrowth of supragingival plaque relative to controls (18). Another recent study found TCS to reduce soft tissue inflammation following scaling and root planing but did not record any significant differences in subgingival microbiota between treatments and controls (19). In contrast, prior work had pointed to both quantitative and qualitative reduction in subgingival microbiota following use of TCS-containing toothpaste, relative to controls (20). Thus, a notable body of literature reported impacts on the human oral microbiome from use of TCS-containing toothpaste for control of inflammatory gum diseases.

Yet it is not necessarily surprising that Poole et al. (2) did not observe any statistically significant effects from exposure to TCS/TCC on the human microbiome structure of the gut and oral cavity. Although Poole et al. (2) performed a substantial and commendable amount of work, the study design was not geared to determine with confidence if and to what extent antimicrobials alter the human microbiome. The authors acknowledge as much themselves when discussing their interesting data on nonsignificant associations found between use of antimicrobial products and body weight changes (2). Whereas small crossover control cohort studies (with, e.g., ≤16 participants [2]) are frequently underpowered for demonstrating with confidence specific human health outcomes, they are still valuable and can be informative. This also applies to the work by Poole et al. (2). Complicating factors in their study included the focus on compounds that are ubiquitous (72% detection frequency for TCS during the non-TCS exposure period), collection of exposure data only for TCS but not for TCC, a high (35%) proportion of out-of-range TCS data requiring use of lower- and upper-bound approximations, uncertainty about the length of time required for the microbiome to return to the baseline, and consideration of long-term outcomes (obesity) that may be ill suited to a study with only a relatively short duration (2).

While presenting a treasure trove of information on the composition and plasticity of the human gut and oral microbiome, the work by Poole et al. (2) does not serve to inform the regulatory decision-making process with respect to antimicrobial compounds. Motivated by a combination of concerns over unwanted environmental and human health impacts and widespread human exposure, and limited or lacking proof of the value of antimicrobials for controlling infectious disease burden in the general population (1), bans or restrictions of the use of TCS or of TCS and TCC have recently been announced in Europe (21), Minnesota (22), and Iowa (23) and are also under consideration for the United States nationwide (24), with a final decision expected from the U.S. Food and Drug Administration (FDA) by September 2016 (1). In addition, a major United States health care provider (25) and multiple international companies (26) have decided to limit use of TCS/TCC in their household product lines.

Whereas usage of TCS and TCC appears to be in decline internationally, as indicated by the aforementioned use restrictions, studies of the human microbiome and interactions between chemicals of daily use and resultant public health impacts (27) are destined to proliferate, thanks to breakthrough developments in high-throughput screening that have compressed analysis times from decades to days. Those who benefit from works such as that conducted by Poole et al. (2) include the scientific community and the general public, with much more still to be learned.
